# A model for optical gain in colloidal nanoplatelets†
†Electronic supplementary information (ESI) available: Synthesis procedure, additional transient absorption spectra and kinetics, fitting procedures, and fitting parameters. See DOI: 10.1039/c7sc04294a


**DOI:** 10.1039/c7sc04294a

**Published:** 2017-11-13

**Authors:** Qiuyang Li, Tianquan Lian

**Affiliations:** a Department of Chemistry , Emory University , 1515 Dickey Drive, NE , Atlanta , GA 30322 , USA . Email: tlian@emory.edu

## Abstract

Cadmium chalcogenide nanoplatelets (NPLs) and their heterostructures have been reported to have low gain thresholds and large gain coefficients, showing great potential for lasing applications. However, the further improvement of the optical gain properties of NPLs is hindered by a lack of models that can account for their optical gain characteristics and predict their dependence on the properties (such as lateral size, concentration, and/or optical density). Herein, we report a systematic study of optical gain (OG) in 4-monolayer thick CdSe NPLs by both transient absorption spectroscopy study of colloidal solutions and amplified spontaneous emission (ASE) measurement of thin films. We showed that comparing samples with the same optical density at the excitation, the OG threshold is not dependent of the NPL lateral area, while the saturation gain amplitude is dependent on the NPL lateral area when comparing samples with the same optical density at the excitation wavelength. Both the OG and ASE thresholds increase with the optical density at the excitation wavelength for samples of the same NPL thickness and lateral area. We proposed an OG model for NPLs that can successfully account for the observed lateral area and optical density dependences. The model reveals that OG originates from stimulated emission from the bi-exciton states and the OG threshold is reached when the average number of excitons per NPL is about half the occupation of the band-edge exciton states. The model can also rationalize the much lower OG thresholds in the NPLs compared to QDs. This work provides a microscopic understanding of the dependence of the OG properties on the morphology of the colloidal nanocrystals and important guidance for the rational optimization of the lasing performance of NPLs and other 1- and 2-dimensional nanocrystals.

## Abstract 2

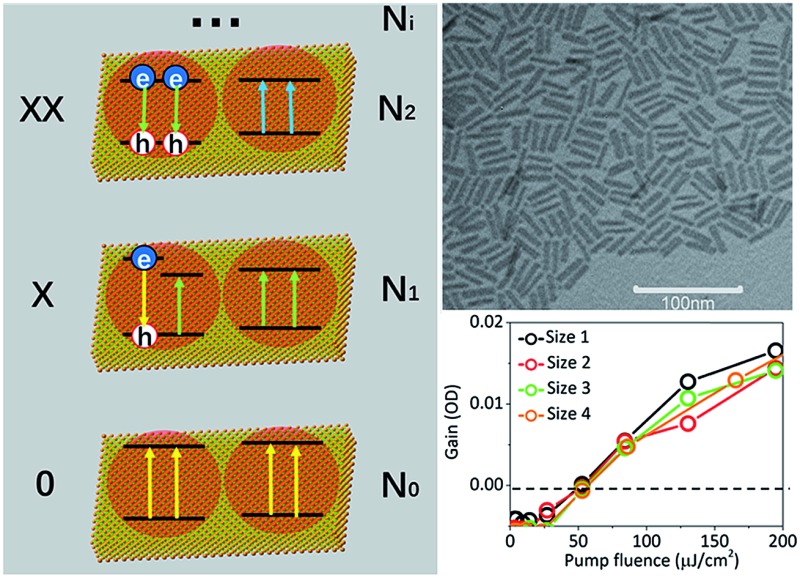
Optical gain in CdSe nanoplatelets is shown to be independent on their lateral size and can be explained by a new optical gain model for 2D nanoplatelets.

## Introduction

Cadmium chalcogenide nanoplatelets (NPLs), CdX (X = Se, S, Te), and their heterostructures have shown many novel properties, such as large absorption cross-sections, uniform 1D quantum confinement, long biexciton Auger lifetimes, and giant oscillator strength.^1^–^14^ These materials have attracted intense interest for lasing applications due to the reported large gain coefficients and low optical gain (OG) threshold.^15^–^25^ For example, the reported threshold of the amplified spontaneous emission (ASE) of the CdSe NPLs is as low as 6 μJ cm^–2^,^15^ which is over an order of magnitude lower than that in cadmium chalcogenide quantum dots (QDs) or QD heterostructures.^26^,^27^ Although OG models for QDs are well understood, it is unclear whether they are applicable to 1D nanorods (NRs) and 2D NPLs because of the fundamental differences in their exciton properties. In 0D QDs, the excitons are confined in all three dimensions, whereas in 2D NPLs and 1D NRs, the excitons are free to move in the plane and along the long axis, respectively, which increases the degree of degeneracy of the band edge exciton states and may alter their gain mechanism. So far, there is not an OG model for 2D NPLs or 1D NRs, and the reasons for the superior OG properties in NPLs remain unclear. In addition, many other interesting differences between the NPLs and QDs may also contribute to their different OG properties. For example, because of the atomically precise thickness, the NPLs have uniform quantum confinement energy and narrow exciton transition linewidth, which should reduce the overlap between the stimulated absorption (loss) and emission (gain) transitions.^3^ It has also been argued that the exciton transition oscillator strength in NPLs may be enhanced by the coherent delocalization of the exciton center of mass in the lateral direction, which should affect the stimulated emission cross section.^3^ The bi-exciton Auger recombination lifetimes in the NPLs are much longer than QDs^6^,^17^ and have been shown to increase linearly with their lateral size.^28^–^30^ It has been proposed that the low OG thresholds in NPLs can be attributed to their longer Auger lifetime.^17^,^19^ These observations would suggest that one of the key differences between NPLs and QDs is the possibility of tuning their OG performance through their lateral size. Olutas *et al.* have reported that the ASE threshold of CdSe NPLs increases with their lateral area.^18^ However, She *et al.* reported a lateral area-independent ASE threshold of the same materials.^19^ These contradictory observations and a lack of understanding of the OG mechanisms in NPLs suggests the need for a systematic study and a model for optical gain in these materials.

Herein, we report a systematic study of the dependence of OG on the lateral area and optical density at pump wavelength in 4 monolayer (ML) CdSe NPLs. We investigate the OG characteristics by femtosecond transient absorption (TA) spectroscopy of the colloidal NPL samples and ASE measurements of the NPL films at room temperature. We show that the OG thresholds are independent of the lateral area of the NPLs, whereas the saturation OG amplitude increases linearly with the area when comparing samples of the same optical density at the excitation wavelength. For NPLs of the same size, their OG and ASE thresholds increase with the optical density at the excitation wavelength. We propose a biexciton gain model that can satisfactorily account for the experimental observations in the NPLs and explain the origin for their much lower OG thresholds compared to QDs. We believe that this model should also be applicable to other 2D nanosheets and 1D nanorods.

## Sample characterization

**Fig. 1:**
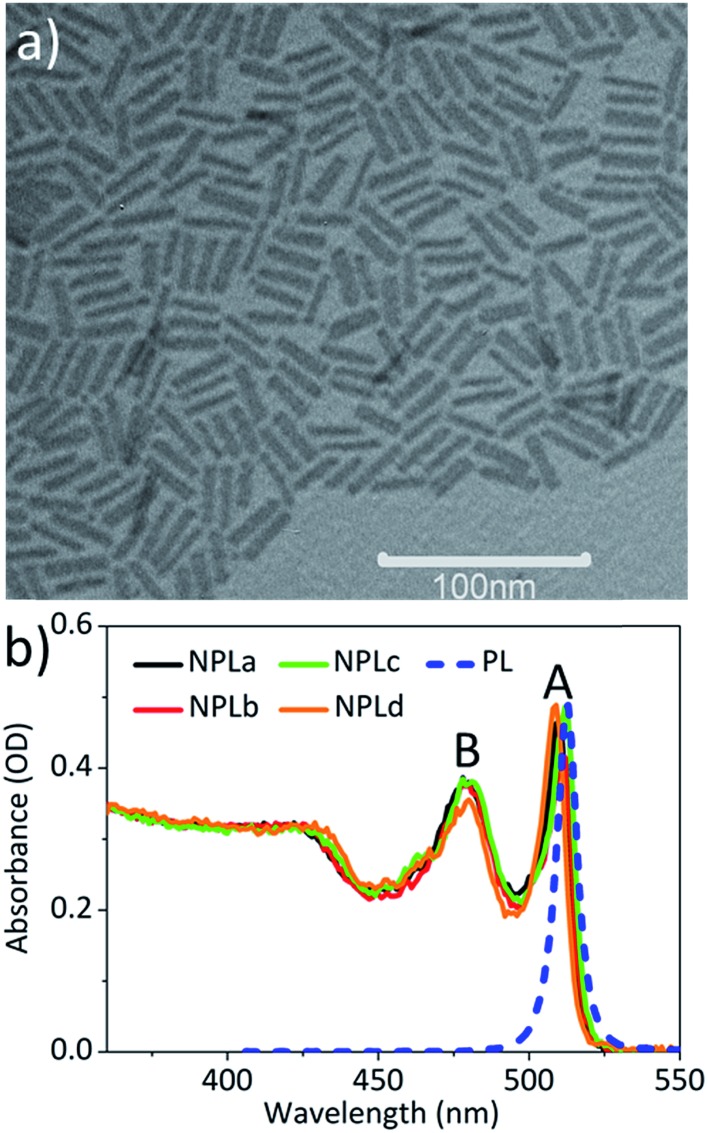
The TEM image and absorption spectra of the CdSe NPLs. (a) The TEM image of NPLd. (b) The absorption spectra of all the CdSe samples of different sizes (NPLa-d) in hexane (solid lines). The blue dashed line is the static photoluminescence spectrum of NPLb.

4 ML CdSe NPLs (with 5 Cd layers, 4 Se layers, and a thickness of ∼1.8 nm) were synthesized according to reported procedures with slight modifications.^3^ The lateral size of the NPLs was tuned by changing the synthesis temperature and reaction time. The detailed synthesis procedures are described in the ESI.† The NPL samples with different lateral sizes are named NPLa to NPLd with increasing lateral size. The same batch of samples have been used in a previous study of the lateral size dependence of the biexciton Auger lifetime in NPLs.^28^Fig. 1a shows the TEM image of NPLd (NPLa to NPLc in Fig. S1†), from which we determined the lateral size of the approximately rectangular shaped NPLs to be from 80.2 ± 12.4 nm^2^ in NPLa to 205.1 ± 35.3 nm^2^ in NPLd (see Table S1†). The absorption spectra of NPLa to NPLd (solid lines, Fig. 1b) show A (∼512 nm) and B (∼480 nm) exciton peaks that correspond to the electron-heavy hole (e-hh) and electron-light hole (e-lh) transitions, respectively. All the NPL samples of different lateral sizes have the same A and B exciton transition energy. The static photoluminescence (PL) spectrum of NPLb (blue dashed line in Fig. 1b) shows a sharp band edge (e-hh) emission peak at ∼518 nm with a full width at half maximum (FWHM) of ∼38 meV. The PL spectra of NPLa to NPLd are compared in Fig. S3,† showing that the band edge emission is independent of the lateral size.

## Lateral area independent optical gain threshold

To determine the optical gain threshold, we carried out a pump fluence dependent TA spectroscopic study of NPLa to NPLd in hexane at room temperature. In a TA measurement, the optical density of the samples under illumination is given by Δ*A*(*λ*,*t*) + *A*_0_(*λ*), where Δ*A*(*λ*,*t*) is the pump-induced absorbance change shown in the transient absorption spectra and *A*_0_(*λ*) is the static absorbance prior to excitation. Thus optical gain is achieved when Δ*A*(*λ*,*t*) + *A*_0_(*λ*) < 0. Because the gain threshold is dependent on the optical density of the sample (see below), to enable the comparison of the NPL samples of different lateral areas, their optical density at pump wavelength (400 nm) was controlled to the same value to ensure the same number of absorbed photons (Fig. 1b). The TA spectra of NPLc at the lowest pump fluence (3 μJ cm^–2^, Fig. S4a†) show long-lived bleach signals of A (∼512 nm) and B (∼480 nm) excitons. According to our previous work on CdSe NPLs, both A and B exciton bleaches can be attributed to state-filling on the first electron level in the conduction band (CB), and the contribution of the hole state-filling in the valence band (VB) is negligible due to degeneracy and strong mixing between the denser hole levels in the VB,^14^,^30^,^31^ similar to cadmium chalcogenide quantum dots and nanorods.^32^–^34^ Fig. S4b† shows the TA spectra of NPLc at the highest pump fluence (629 μJ cm^–2^) when the bleach amplitudes of the A and B exciton states at an early delay time have saturated. Compared to those at low pump fluence, these spectra show an additional broad negative peak, Δ*A*(*λ*,*t*) < 0, at energy lower than the A exciton (∼520–560 nm), which can be attributed to the optical gain (OG) signal,^19^ similar to that reported in CdSe quantum dots (QDs).^27^

**Fig. 2:**
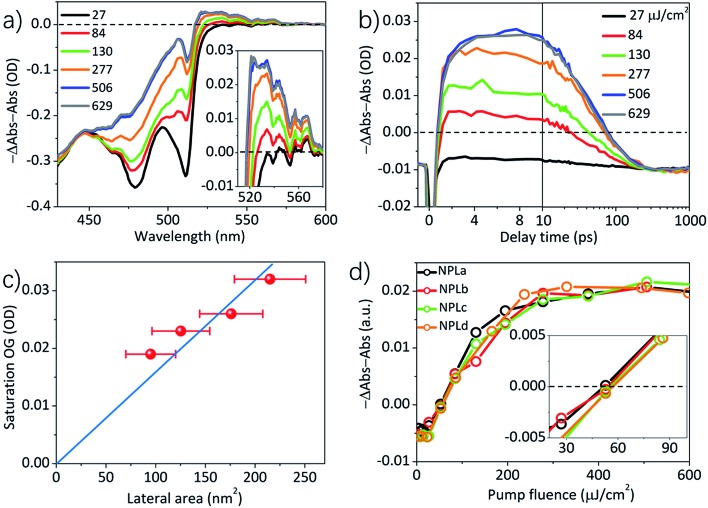
The lateral area independent optical gain thresholds. (a) The gain spectra, –(Δ*A*(*λ*,*t*) + *A*_0_(*λ*)), of NPLc at 3–4 ps at indicated fluences of 400 nm excitation. (b) The OG kinetics (probed at 528 nm) of NPLc at different pump fluences. (c) The saturation OG amplitudes (at 3–4 ps) of different NPL samples as a function of the lateral area. (d) The normalized OG amplitude (at 3–4 ps) of all the NPL samples as a function of the pump fluences. The inset shows the OG amplitude at low pump fluence (20–100 μJ cm^–2^), where the intercept on the *x*-axis gives the OG threshold.

The gain spectra (–Δ*A*(*λ*,*t*) – *A*_0_(*λ*)) at 3–4 ps of NPLc at different pump fluences are shown in Fig. 2a and an expanded view of the gain spectra (Fig. 2a inset) shows a broad OG peak with a maximum at ∼528 nm. The kinetics of the gain signal of NPLc at the OG peak wavelength (∼528 nm) and with different pump fluences are compared in Fig. 2b. All the kinetics show a negative signal around time zero (<1 ps), which reflects a red-shifted exciton absorption caused by exciton–exciton interaction.^14^,^20^,^28^–^31^ After 1 ps, the OG amplitude remains negative, indicating no OG, for pump fluences below 27 μJ cm^–2^. The OG amplitude increases with increasing pump fluence and becomes positive, indicating gain, after a pump fluence of 84 μJ cm^–2^. The OG amplitude saturates after the pump fluence reaches 506 μJ cm^–2^. NPL samples with different lateral areas were also studied and their OG kinetics at ∼528 nm are shown in Fig. S4.†

As shown in Fig. 2b and S4,† the OG amplitude of all the samples reaches the highest value at a delay time of 3–4 ps, after which the OG signals decay due to multiple exciton Auger recombination.^28^ A plot of the maximum OG amplitudes (at 3–4 ps) as a function of the pump fluence (Fig. S7b†) shows that for all the NPL samples, the OG reaches saturation at high pump fluences, but the saturation OG value increases linearly with lateral area (Fig. 2c). To facilitate comparison of the gain threshold, we have scaled the OG of different samples to the same saturation amplitude and plotted the normalized OG as a function of the pump fluence in Fig. 2d. The comparison shows that the normalized OG of all the NPL samples exhibits the same dependence on the pump fluence: a linear increase of OG with pump fluence between 15–150 μJ cm^–2^ and reaching saturation between 150–500 μJ cm^–2^. As shown in the inset of Fig. 2d, the intercept of the OG amplitude on the *x*-axis yields the same OG threshold of 54.6 ± 1.8 μJ cm^–2^ for all the four samples under our experimental conditions (optical density of 0.31 ± 0.01 at 400 nm pump), independent of their lateral area.

## Optical density dependent OG threshold

**Fig. 3:**
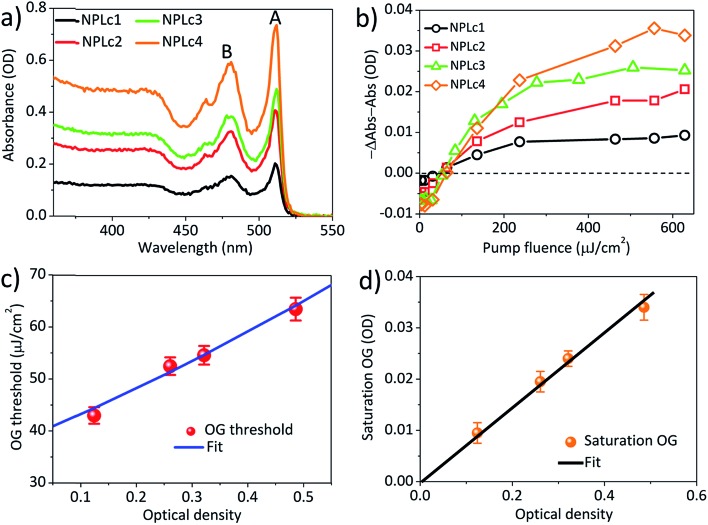
The optical density dependent optical gain threshold. (a) Absorption spectra of NPLc samples with different concentrations (and optical densities at 400 nm). (b) The OG amplitudes as a function of the pump fluence for the NPLc samples with different concentrations. (c) The OG thresholds as a function of the NPLc optical density at pump wavelength (400 nm). The blue solid line is the fit according to the OG threshold model described in the main text. (d) The saturation OG amplitude as a function of the NPLc optical density at 400 nm. The black line is the linear fit.

To investigate how the OG threshold changes with sample optical density, we carried out TA study of the NPLc samples in hexane solution with different concentrations, named NPLc1 to NPLc4 in the order of increasing NPL concentration (NPLc3 is the sample used in Fig. 2). The absorption spectra of NPLc1 to NPLc4 (Fig. 3a) show that the optical density at 400 nm increases from 0.12 to 0.49 from sample NPLc1 to NPLc4. These samples were investigated using the same pump fluence dependent TA measurement and analysis method described above. Their OG kinetics as a function of the pump fluence are shown in Fig. S4.† Their peak OG amplitudes at 3–4 ps and ∼528 nm are plotted as a function of the pump fluence in Fig. 3b. The intercept of these data on the *x*-axis yields OG thresholds of 43.0 ± 1.6, 52.5 ± 1.7, 54.6 ± 1.8, and 63.5 ± 2.2 μJ cm^–2^ for NPLc1 to NPLc4, respectively. As shown in Fig. 3c, these OG threshold values increase with the optical density at pump wavelength (400 nm). Fig. 3d shows that the saturation OG amplitude increases linearly with optical density at 400 nm, indicating more gain at saturation if more photons are absorbed. Similar optical density dependent ASE thresholds using NPLc films prepared by spin-coating of NPLc solutions with different concentrations on a glass substrate were also observed (Fig. S5†).

## Model of the optical gain threshold

**Fig. 4:**
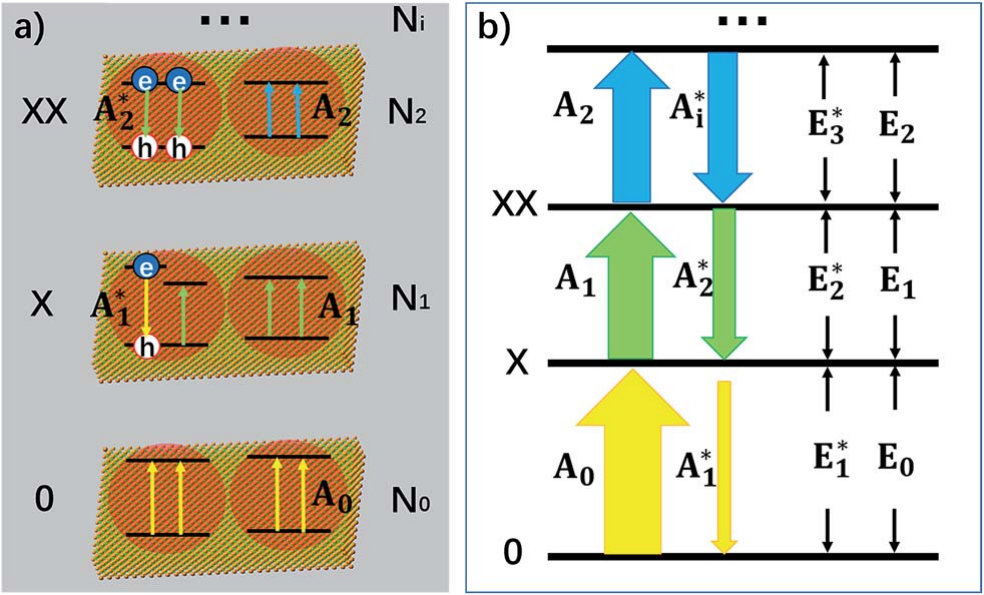
A model for biexciton optical gain in colloidal CdSe NPLs. The relevant states and transitions in (a) single particle (electron or hole) and (b) exciton state representations, respectively. N_0_ (0), N_1_ (X) and N_2_ (XX) are the population probabilities (and label) of the NPL states with 0, 1 and 2 excitons, respectively. The colors of the arrows indicate the transition energies of the stimulated absorptions (upward arrow, *A*_*i*_) and emissions (downward arrow, 
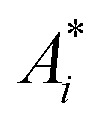
) from state *i*; (a) shows example NPLs that can contain up to 4 conduction band edge excitons (*N*_s_ = 4).

To explain the experimental results described above, we propose a model for OG in NPLs. The details of this OG model can be found in the ESI† and only the key aspects are summarized here. This model is an extension of the previous gain model proposed for QDs,^26^ which, because of the confinement in all three dimensions, can only accommodate two band edge excitons. In this model, we assume that because of the large (unconfined) lateral dimension of NPLs, the number (*N*_s_) of band edge (or A) excitons can exceed 2, increasing the complexity of the number of transitions associated with single and multiple band edge exciton states, as shown in Fig. 4 (for an example of *N*_s_ = 4). This assumption is based on our previous observations of NPLs,^20^ and the 2D hydrogen-like exciton model in 2D structures.^35^,^36^ On the basis of the redshift of OG and ASE from NPL emission, it has been proposed that OG or ASE in NPLs can be attributed to stimulated emission from band edge bi-exciton states,^17^–^19^ similar to QDs.^27^ Therefore, our model only considers band edge exciton states with 0, 1, … *N*_s_ band edge excitons, which are labeled as 0, X, XX, … states, respectively, and their population probabilities are indicated by *N*_*i*_ (*i* = 0 to *N*_s_). Each exciton state (*i*) can undergo stimulated absorption (upward arrows in Fig. 4) or emission (downward arrows in Fig. 4) with partial cross-sections per NPL of *A*_*i*_ (*i* = 0 to *N*_s_–1) and 
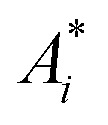
 (*i* = 1 to *N*_s_), respectively, given by eqn (1) and (2):1
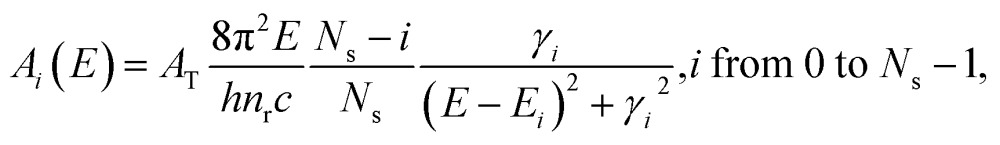

2
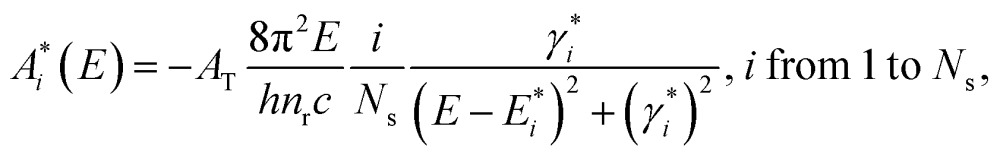



In eqn (1) and (2), *h* is the Planck’s constant, *n*_r_ is the refractive index, and c is the speed of light. *A*_T_ is the transition strength of the band edge excitons (e-hh) per NPL, which is proportional to the NPL lateral area, *A*_QW_. 2*γ*_*i*_ and 
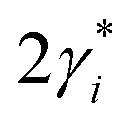
 are the full width at half maximum of the absorption and emission spectra of the *N*_*i*_ species, respectively. *E*_*i*_ and 
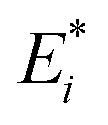
 are the stimulated absorption and emission peak energy for the *N*_i_ species, respectively. We set both *γ*_0_ and 
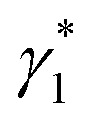
 to ∼19 meV for both single band edge exciton absorption and emission according to Fig. 1b. We assume both *γ*_*i*–1_ and 
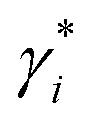
 (*i* from 2 to *N*_s_) to be the same as the broad OG spectra (∼50 meV) shown in Fig. 2a. *E*_0_ and 
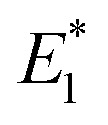
 are 2.42 eV and 2.39 eV, respectively, according to the A exciton absorption (512 nm) and emission (518 nm) wavelengths in Fig. 1b. The energy of bi-exciton absorption (*E*_1_) and emission 
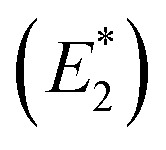
 is assumed to be the optical gain energy, *E*_OG_, which is set to the OG peak value (2.35 eV, 528 nm) according to Fig. 2a. The inhomogeneous distribution of *E*_OG_ is ignored due to the uniform 1D quantum confinement of the NPLs. The shift (from *E*_OG_) of the transition energies for the tri and higher exciton states is assumed to be much smaller than the transition line width: 
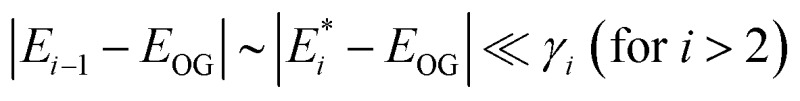
. This assumption is based on the broad transition width for the tri and higher exciton states and Coulomb screening of the multi-excitons reported in other 2D materials,^37^ although these values have not been observed directly in our NPLs.

The absorption coefficient of the NPL ensemble at OG energy is:3
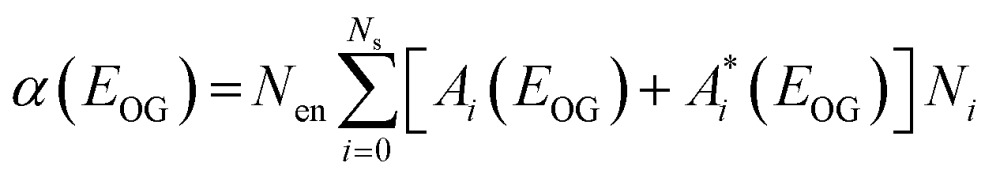
where *N*_en_ is the number of NPLs in the ensemble, which is proportional to the NPL molar concentration, *C*_m_. The population probability of the NPL species (*N*_*i*_) is assumed to follow Poisson distribution: 
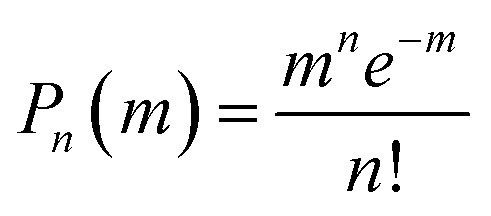
, which represents the possibility of finding NPLs with n excitons when the average number of excitons per NPL is m. The optical gain threshold is achieved when *α*(*E*_OG_) = 0. Solving eqn (3) numerically under this condition leads to *m*_th_(*N*_s_), the average number of excitons per NPL at the OG threshold, of 0.49 (±0.01) *N*_s_ (see Fig. S6 and Table S2†). The result suggests that OG is reached when about half of the band edge exciton states are occupied. Under this condition, the gain (emission from excited states) equals the loss (absorption from ground states). Within the limits of QDs (*N*_s_ = 2), *m*_th_ ∼ 1, which is consistent with previous findings on QDs.^26^

Because *m* is proportional to the pump fluence (*I*) and the optical density at pump wavelength following Beer’s law, the *m*_th_ value can be converted to threshold pump fluence, *i.e.* the OG threshold (*I*_th_), according to eqn (4).4
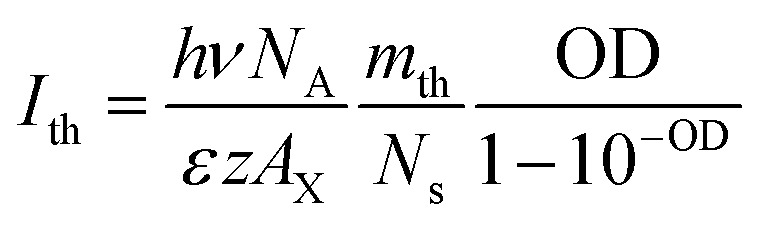



In eqn (4), OD = *εzA*_QW_*C*_m_*L*, *hν* is the pump photon energy (3.1 eV), *N*_A_ is Avogadro’s constant, *A*_X_ = *A*_QW_/*N*_s_, *ε* is the molar absorption coefficient per unit NPL volume, *z* is the NPL thickness (∼1.8 nm), *L* is the light path of the cuvette (1 mm) and *m*_th_/*N*_s_ = 0.49 ± 0.01. The details of the derivation can be found in the ESI.† According to eqn (4), when comparing NPL samples of the same thickness, their OG thresholds are independent of the NPL lateral area as long as the optical densities at pump wavelength are the same. This prediction is consistent with the experimental result shown in Fig. 2d. Moreover, the observed OD dependent OG and ASE thresholds can be well fitted by eqn (4), as shown in Fig. 3c and S5f†, respectively, providing further support for our OG model.

At the limit of large *m*, the optical gain reached saturation with the gain amplitude given by eqn (5).5
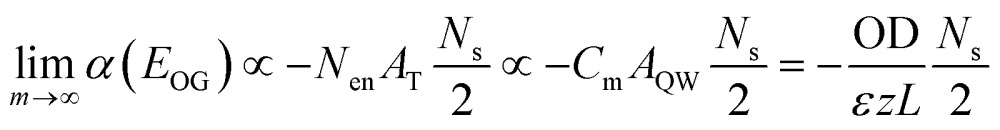



This predicts that the saturation gain amplitude increases linearly with both the lateral area (proportional to *N*_s_) and optical density (OD) of the NPL (Fig. S7a†), both of which are consistent with the experimental findings as shown in Fig. 2c and 3d, respectively.

Finally, our model (eqn (3)) also predicts how OG increases with the pump fluence. The observed OG amplitude as a function of *m* can be reasonably well fitted by our model (Fig. S7h†), although the simulated OG saturates at a lower value of *m* compared to the experimental results for NPLs with large *N*_s_ (*N*_s_ > 3). The origin of this deviation is not well understood, but it indicates that some loss factors are not fully accounted for in our model. This is likely due to the lack of consideration of a transition width distribution from higher exciton states in our model, which have not been experimentally observed.

There have been two contradicting reports on whether the ASE threshold depends on the NPL lateral area.^18^,^19^ In ref. 19, the optical density at pump wavelength of different NPL samples was controlled to similar values, and the lateral area-independent ASE thresholds were observed,^19^ which is consistent with our experimental results and OG model. In ref. 18, the lateral area dependent ASE threshold was observed, but it is unclear whether the optical density at pump wavelength for samples of different NPL areas was controlled to the same values.^18^

Our result suggests that optical gain is achieved when the average number of excitons per NPL is close to half (0.49) of the band edge exciton states, which is similar to the OG requirement in QDs. Despite this similarity, the optical gain thresholds in QDs have been reported to be more than an order of magnitude higher than those in NPLs.^15^,^26^,^27^ According to our model, the lower OG threshold of the NPLs can be attributed to the following reasons. First, the intrinsic absorption cross section of NPLs, *i.e.* the absorption coefficient per unit volume (*ε*), is larger than that of QDs, which according to eqn (4) leads to lower OG threshold. Recently, Achtstein *et al.* have reported that the intrinsic absorption coefficient of CdSe NPLs is over 3 folds larger than that in CdSe QDs due to the larger aspect ratio of NPLs.^38^ Second, the ratio of biexciton binding energy (∼40 meV) and transition linewidth (∼38 meV), 
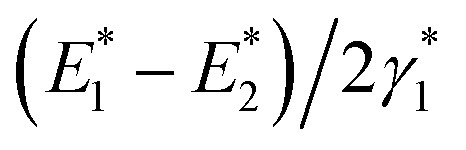
, in NPLs (∼1.0) is larger than that in QDs (<0.3) whose biexciton binding energy is <30 meV and transition linewidth is ∼100 meV.^26^ This reduces the overlap between the gain and loss transitions, decreasing the OG threshold in NPLs. The latter can be attributed to the atomically precise uniform thickness of NPLs, which reduces the inhomogeneous broadening of the exciton transition energy. Such sharp transitions are difficult to achieve in QDs because of the broad size distribution and large inhomogeneous distribution of transition energies. In addition, the symmetry of the NPLs dictates that both the electric field of the exciton and the dipole moments lie within the lateral plane,^39^ which may account for the observed large Stark effect induced shift of transition energy between the bi-exciton and single exciton states.

## Conclusions

In summary, we have systematically studied the dependence of the OG properties of CdSe NPLs on their lateral area and the optical density at pump wavelength using TA spectroscopy and ASE measurements. We show that the OG threshold is lateral area independent when comparing samples of the same optical density at the excitation wavelength, although the saturation OG amplitude increases with the lateral area. Furthermore, for samples of the same NPL size, the OG and ASE threshold increases with their optical density at pump wavelength. To account for these observations, we proposed an optical gain model for 2D CdSe NPLs. This model assumes that the number of band edge excitons scales with the NPL lateral area (and can exceed 2) and optical gain results from the stimulated emission from biexciton states. Our model successfully explains the experimental observations. The model also reveals that OG is achieved when the average number of excitons reaches ∼49% of the band edge exciton states. This OG requirement is similar to that in QDs, despite the observed OG threshold in NPLs being an order of magnitude smaller than that in QDs. According to our model, the lower OG threshold of NPLs can be attributed to their unique 2D morphology, which leads to a larger intrinsic absorption coefficient, narrower transition linewidth, and larger shift between the bi- and single-exciton state. This work provides not only important insights on how the crystal morphology affects the OG properties of the colloidal nanocrystals, but also guidance on the rational improvement of the OG and ASE in NPL materials for lasing applications. Finally, we believe that this OG model should be applicable to other 2D and 1D nanocrystals.

## Conflicts of interest

There are no conflicts to declare.

## Supplementary Material

Supplementary information

## References

[cit1] Ithurria S., Dubertret B. (2008). J. Am. Chem. Soc..

[cit2] Ithurria S., Bousquet G., Dubertret B. (2011). J. Am. Chem. Soc..

[cit3] Ithurria S., Tessier M. D., Mahler B., Lobo R. P. S. M., Dubertret B., Efros A. (2011). Nat. Mater..

[cit4] Achtstein A. W., Schliwa A., Prudnikau A., Hardzei M., Artemyev M. V., Thomsen C., Woggon U. (2012). Nano Lett..

[cit5] Ithurria S., Talapin D. V. (2012). J. Am. Chem. Soc..

[cit6] Kunneman L. T., Tessier M. D., Heuclin H., Dubertret B., Aulin Y. V., Grozema F. C., Schins J. M., Siebbeles L. D. A. (2013). J. Phys. Chem. Lett..

[cit7] Tessier M. D., Mahler B., Nadal B., Heuclin H., Pedetti S., Dubertret B. (2013). Nano Lett..

[cit8] Tessier M. D., Spinicelli P., Dupont D., Patriarche G., Ithurria S., Dubertret B. (2014). Nano Lett..

[cit9] Benchamekh R., Gippius N. A., Even J., Nestoklon M. O., Jancu J. M., Ithurria S., Dubertret B., Efros A. L., Voisin P. (2014). Phys. Rev. B: Condens. Matter Mater. Phys..

[cit10] Pedetti S., Ithurria S., Heuclin H., Patriarche G., Dubertret B. (2014). J. Am. Chem. Soc..

[cit11] Antanovich A. V., Prudnikau A. V., Melnikau D., Rakovich Y. P., Chuvilin A., Woggon U., Achtstein A. W., Artemyev M. V. (2015). Nanoscale.

[cit12] Delikanli S., Guzelturk B., Hernández-Martínez P. L., Erdem T., Kelestemur Y., Olutas M., Akgul M. Z., Demir H. V. (2015). Adv. Funct. Mater..

[cit13] Achtstein A. W., Scott R., Kickhöfel S., Jagsch S. T., Christodoulou S., Bertrand G. H. V., Prudnikau A. V., Antanovich A., Artemyev M., Moreels I., Schliwa A., Woggon U. (2016). Phys. Rev. Lett..

[cit14] Wu K., Li Q., Jia Y., McBride J. R., Xie Z.-x., Lian T. (2015). ACS Nano.

[cit15] Grim J. Q., Christodoulou S., Di Stasio F., Krahne R., Cingolani R., Manna L., Moreels I. (2014). Nat. Nanotechnol..

[cit16] Guzelturk B., Kelestemur Y., Olutas M., Delikanli S., Demir H. V. (2014). ACS Nano.

[cit17] She C. X., Fedin I., Dolzhnikov D. S., Demortiere A., Schaller R. D., Pelton M., Talapin D. V. (2014). Nano Lett..

[cit18] Olutas M., Guzelturk B., Kelestemur Y., Yeltik A., Delikanli S., Demir H. V. (2015). ACS Nano.

[cit19] She C., Fedin I., Dolzhnikov D. S., Dahlberg P. D., Engel G. S., Schaller R. D., Talapin D. V. (2015). ACS Nano.

[cit20] Li Q., Xu Z., McBride J. R., Lian T. (2017). ACS Nano.

[cit21] Yang Z., Pelton M., Fedin I., Talapin D. V., Waks E. (2017). Nat. Commun..

[cit22] Li M., Zhi M., Zhu H., Wu W.-Y., Xu Q.-H., Jhon M. H., Chan Y. (2015). Nat. Commun..

[cit23] Diroll B. T., Talapin D. V., Schaller R. D. (2017). ACS Photonics.

[cit24] Kelestemur Y., Dede D., Gungor K., Usanmaz C. F., Erdem O., Demir H. V. (2017). Chem. Mater..

[cit25] Kelestemur Y., Guzelturk B., Erdem O., Olutas M., Gungor K., Demir H. V. (2016). Adv. Funct. Mater..

[cit26] Klimov V. I., Ivanov S. A., Nanda J., Achermann M., Bezel I., McGuire J. A., Piryatinski A. (2007). Nature.

[cit27] Klimov V., Mikhailovsky A., Xu S., Malko A., Hollingsworth J., Leatherdale C., Eisler H. J., Bawendi M. (2000). Science.

[cit28] Li Q., Lian T. (2017). Nano Lett..

[cit29] Li Q., Zhou B., McBride J. R., Lian T. (2017). ACS Energy Lett..

[cit30] Li Q., Wu K., Chen J., Chen Z., McBride J. R., Lian T. (2016). ACS Nano.

[cit31] Wu K., Li Q., Du Y., Chen Z., Lian T. (2015). Chem. Sci..

[cit32] Klimov V. I. (2000). J. Phys. Chem. B.

[cit33] Zhu H. M., Lian T. Q. (2012). J. Am. Chem. Soc..

[cit34] Wu K. F., Zhu H. M., Liu Z., Rodriguez-Cordoba W., Lian T. Q. (2012). J. Am. Chem. Soc..

[cit35] Shinada M., Sugano S. (1966). J. Phys. Soc. Jpn..

[cit36] Schmitt-Rink S., Chemla D. S., Miller D. A. B. (1985). Phys. Rev. B: Condens. Matter Mater. Phys..

[cit37] Chernikov A., Ruppert C., Hill H. M., Rigosi A. F., Heinz T. F. (2015). Nat. Photonics.

[cit38] Achtstein A. W., Antanovich A., Prudnikau A., Scott R., Woggon U., Artemyev M. (2015). J. Phys. Chem. C.

[cit39] Gao Y., Weidman M. C., Tisdale W. A. (2017). Nano Lett..

