# Supplementary material for: A model for optical gain in colloidal nanoplatelets
Source: Chem Sci. 2017 Nov 13;9(3):728–34. doi: 10.1039/c7sc04294a (PMC5870475; doi:10.1039/c7sc04294a)
Supplement: Supplementary file 1 [file SC-009-C7SC04294A-s001.pdf]

Electronic Supplementary Material (ESI) for Chemical Science.
This journal is © The Royal Society of Chemistry 2017

Electronic Supplementary Information for:

A Model for Optical Gain in Colloidal Nano-platelets

Qiuyang Li, Tianquan Lian*

Department of Chemistry, Emory University, 1515 Dickey Drive, NE, Atlanta, GA, 30322, USA

Content list:

S1. Sample synthesis

S2. The TEM images and lateral size of samples

S3. Experimental setup

S4. The static photoluminescence spectra

S5. Transient absorption spectra and optical gain kinetics

S6. The amplified spontaneous emission measurements

S7. Optical gain mechanisms and threshold model

S1. Sample synthesis

Synthesis of 4 mono-layered CdSe Colloidal nanoplatelets (NPLs): CdSe colloidal NPLs were synthesized following the procedures reported in the literatures with slight modifications.[1] 170 mg Cadmium myristate ($Cd(myr)_2$) and 14 mL of 1-Octadecene (ODE) were introduced to a 25 mL three-neck flask. After degassed under vacuum at 100 °C for 1 hour, 12 mg Se powder in 1 mL ODE were injected, which is followed by heating sample under argon flow. When the temperature reached 205 °C, 40 mg Cadmium acetate dihydrate ($Cd(Ac)_2{\cdot}2H_2O$) was introduced into the solution while being heated. The NPLa was obtained by stopping the reaction when the temperature reached 230 °C. The NPLb to NPLd were obtained by keeping the reaction at 240 °C for 1 min, 5 min, and 15 min, respectively. The reaction was stopped by submerging the flask into a water bath and by the injection of 1 mL oleic acid (OA) into the solution. The products were a mixture of CdSe NPLs and quantum dots (QDs) and the CdSe NPLs were precipitated by adding ethanol and centrifugation at 5000 rpm for 3 min. The final NPL products were dispersed in hexane.

Preparation of CdSe NPL films: NPL films used in amplified spontaneous emission (ASE) measurement were prepared using spin-coating method following the reported procedures with slight modicications.[2] The as-prepared CdSe NPLs were precipitated by ethanol to remove additional ligands (OA) in NPL solutions. NPLs was then dispersed in 4:1 (v:v) hexane/octane mixture and deposited on a glass substrate by spin-coating in air

S2. The TEM images and lateral size of samples

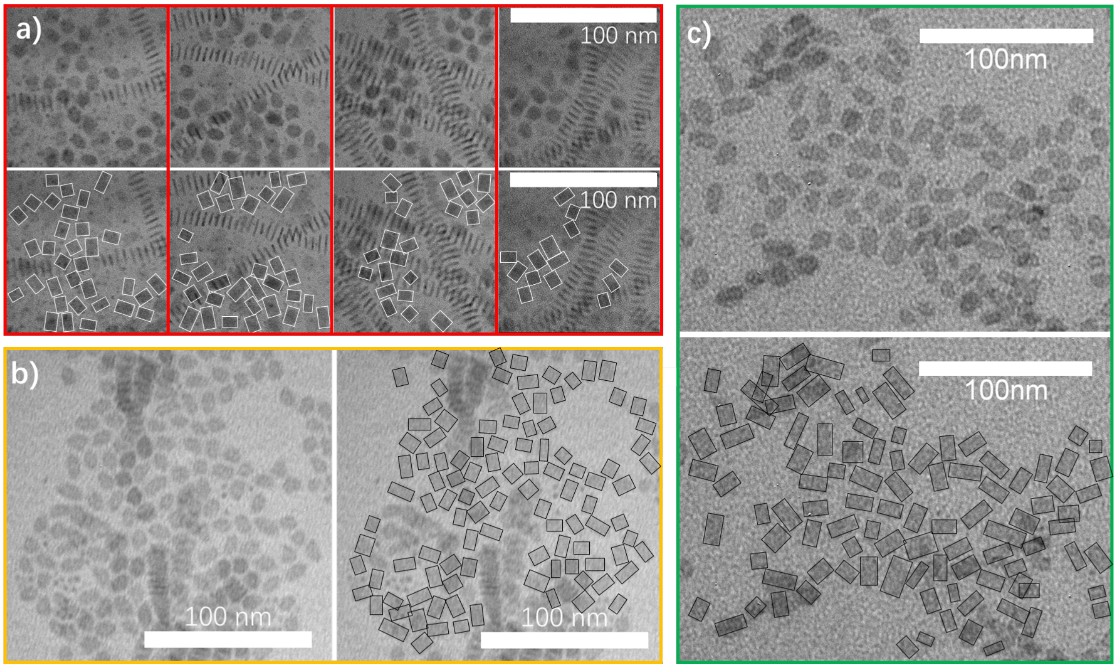

Figure S1. TEM images of (a) NPLa, (b) NPLb, and (c) NPLc. The length and width of all samples are determined by the solid lines marked in lower panels of (a) for NPLa, right panel of (b) for NPLb, and lower panel of (c) for NPLc.

The length, width, and area of all CdSe colloidal NPL samples are measured according to Figure S1 (NPLa to NPLc) and Figure 1a (NPLd). The distribution of NPL length and width is shown in Figure S2. The average length, width, and area with the errors are listed in Table S1.

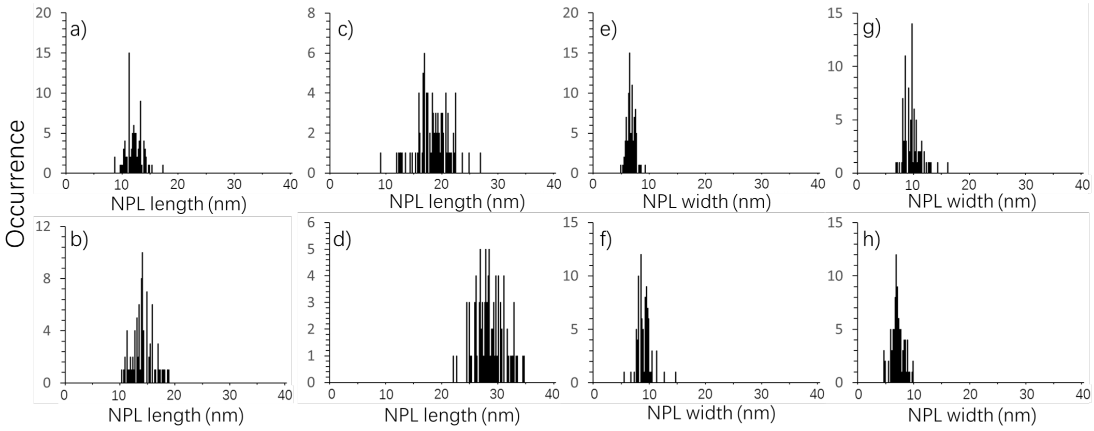

Figure S2. The histograms for NPL length of (a) NPLa, (b) NPLb, (c) NPLc, (d) NPLd, and NPL width of (e) NPLa, (f) NPLb, (g) NPLc, and (h) NPLd.

Table S1. The length, width, and area with errors of CdSe colloidal NPLs

	Length/nm	Width/nm	Area/nm^2
NPLa	11.8±1.4	6.8±0.7	80.2±12.4
NPLb	14.0±1.9	8.8±1.2	123.2±23.8
NPLc	18.2±3.0	9.5±1.5	172.9±35.6
NPLd	29.3±2.6	7.0±1.1	205.1±35.3

S3. Experimental set-up

Femtosecond Transient Absorption (TA) Set-up: Femtosecond transient absorption measurements were conducted at room temperature in a Helios spectrometer (Ultrafast Systems LLC) with pump and probe beams derived from an amplified Ti: sapphire laser system (Coherent Legend, 800 nm, 150 fs, 2.4 mJ/pulse, and 1 kHz repetition rate). The 800 nm output pulse was frequency-doubled to produce the 400 nm (3.1 eV) pump beam. A series of neutral-density filter wheels were used to adjust the power the pump beam. The pump beam was focused at the sample with a beam waist of about 300 μm. A white light continuum (WLC) from 430 to 850 nm (1.5 to 2.9 eV) was generated by attenuating and focusing ~10 μJ of the 800 nm pulse into a Ti: sapphire window. The WLC

was split into a probe and reference beam. The probe beam was focused with an Al parabolic reflector onto the sample (with a beam waist of 150 μm at the sample). The reference and probe beams were focused into a fiber-coupled multichannel spectrometer with complementary metal-oxide-semiconductor (CMOS) sensors and detected at a frequency of 1 kHz. The intensities of the pump and probe beams were tuned to correct for pulse-to-pulse fluctuation of the white-light continuum. The delay between the pump and probe pulses was controlled by a motorized delay stage. The pump beam was chopped by a synchronized chopper to 500 Hz. The change in absorbance for the pumped and un-pumped samples was calculated. 1 mm cuvettes were used for all solution sample TA spectroscopy measurements. The instrument response function (IRF) of this system was measured to be ~150 fs by measuring solvent responses under the same experimental conditions (with the exception of a higher excitation power).

Static photoluminescence (PL) set-up: all static PL measurements were performed with FluoroMax-3 Spectrofluorometer of HORIBA Scientific at room temperature. 1 cm cuvettes were used for all solution sample measurements.

Amplified Spontaneous Emission (ASE) measurement: all ASE measurements were conducted at room temperature in air. The NPL film was pumped with the same 400 nm pulses described in TA set-up. The pump beam was focused into a stripe along the NPL films by a cylindrical lens. The length of the stripe was determined to be the same as the beam size before focusing (2.2 mm) and the width of the stripe was determined to be 32 μm using the knife-edge technique. The emission was detected at the edge of NPL films along the stripe direction by a USB spectrometer (HR2000+, Ocean Optics).

S4. The static photoluminescence spectra

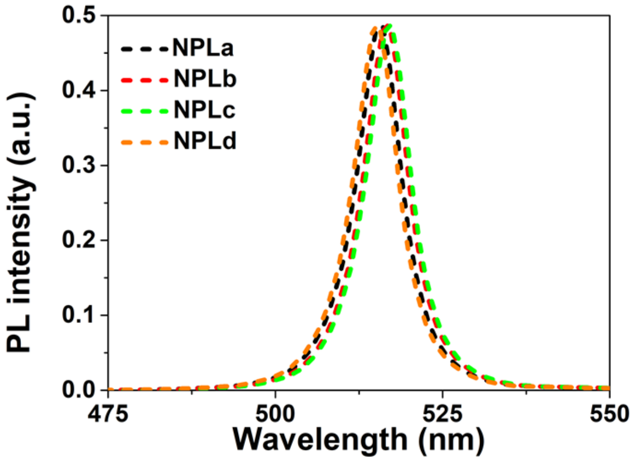

Figure S3. Static PL spectra of all CdSe NPL samples.

S5. Transient absorption spectra and optical gain kinetics

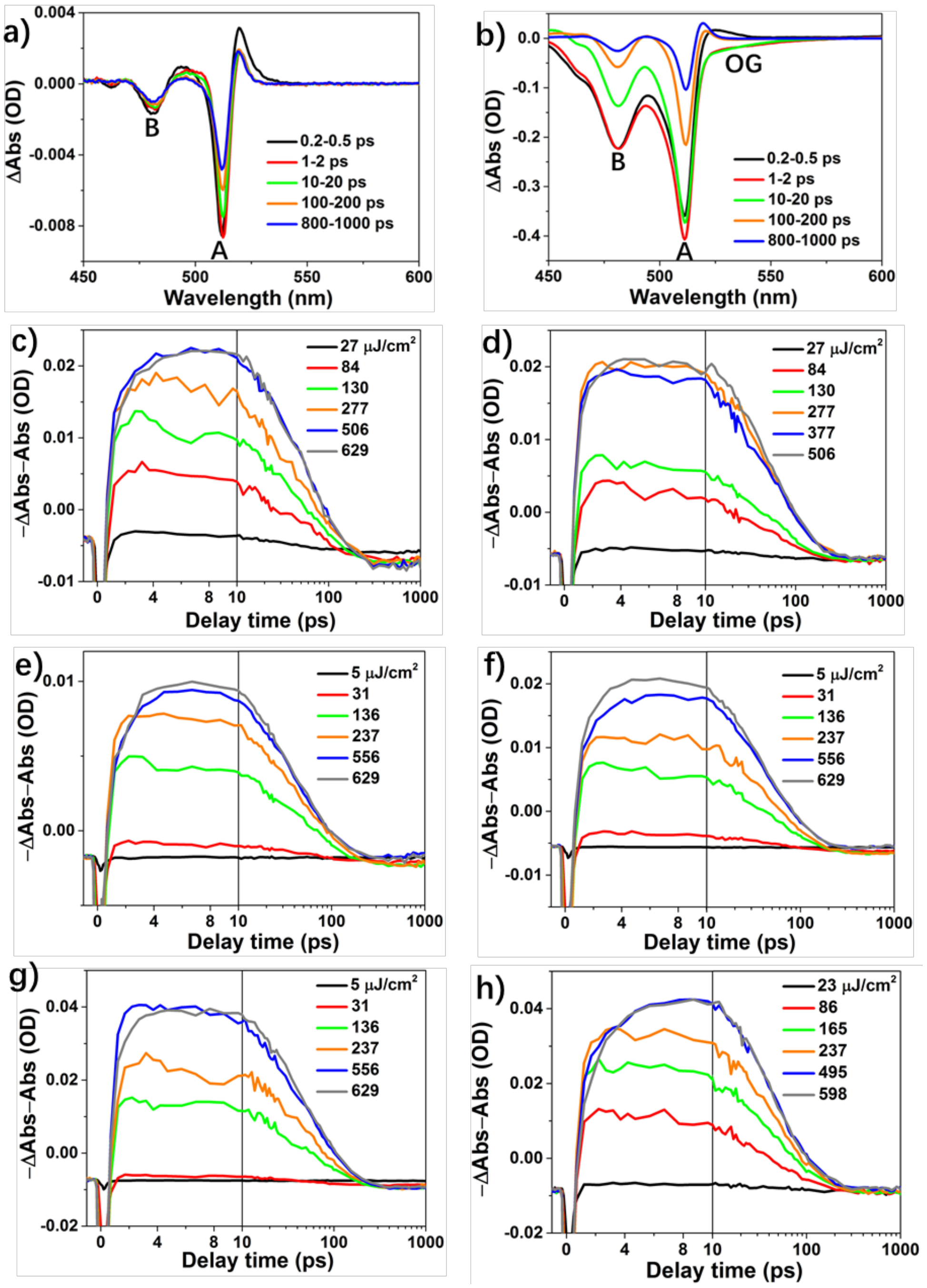

Figure S4. TA spectra of NPLc at indicated delay times after 400 nm pump measured at a pump fluence of (a) 3 μJ/cm^2 and (b) 629 μJ/cm^2. Optical gain kinetics at ~528 nm at different pump fluences of (c) NPLa, (d) NPLb, (e) NPLd, (f) NPLc1, (g) NPLc2, and (h) NPLc4.

S6. Amplified spontaneous emission measurements

As another way of characterizing potential laser performance of NPLs, we also studied how ASE threshold depends on the optical density of the sample. For this study, three NPLc films, named Film1 to Film3 with increasing absorbance (Figure S5a), were prepared by spin-coating NPLc solution with increasing concentrations on a glass substrate. ASE measurement was carried out using the same 400 nm pulse excitation that was also used for the TA measurement. The pump beam was focused into a stripe along the film plane using a cylindrical lens. The emission was detected at the film edge along the stripe direction. The emission spectra of Film1 to Film3 as a function of fluence are shown in Figure S5b to S5d, respectively. The emission spectra can be assigned to ASE, consistent with previous reports.[2, 3] Unlike previous reports, no single exciton band edge emission, which is centered at ~518 nm, was observed in the ASE measurement. This likely indicates that single exciton band edge emission intensity is too small to be observed under our experimental conditions. As shown in Figure S5e, the ASE peak intensity of all films increases linearly with pump fluence within 1 mJ/cm^2. The intercepts on the x-axis of the linear fits of these data reveal ASE thresholds of 44.8 ± 4.0, 50.0 ± 4.7, and 112.4 ± 4.2 μJ/cm^2 for Film1 to Film3, respectively. As shown in Figure S5f, the ASE threshold shows the same dependence on NPL optical density as the OG threshold: it increases in films of higher optical density. Furthermore, the slope of the linear increase of ASE with pump fluence increases with the optical density at the excitation wavelength, also similar to OG threshold.

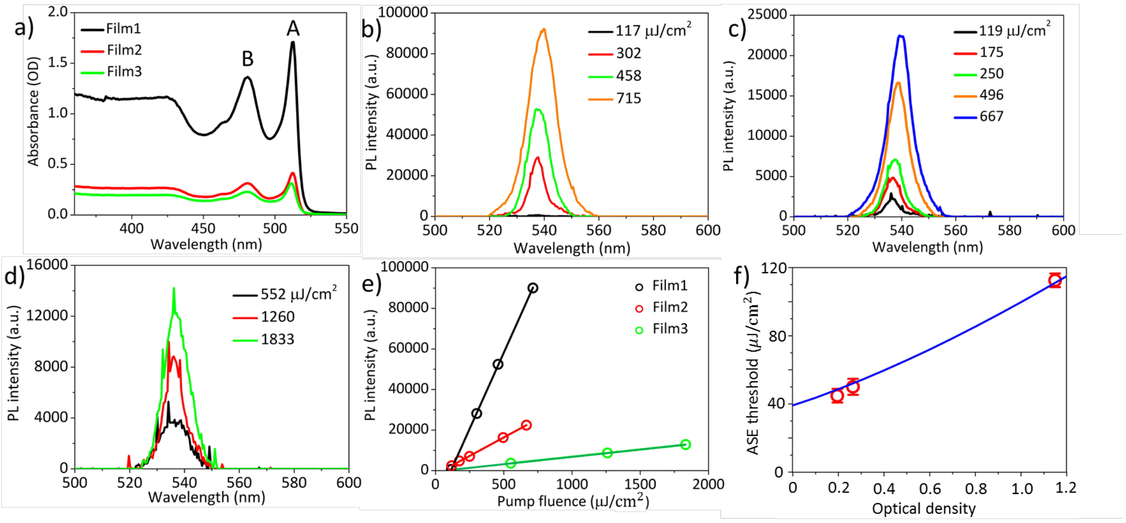

Figure S5. ASE in NPL films. (a) Absorption spectra of different NPLc films. ASE spectra of NPLc (b) Film1, (c) Film2, and (d) Film3 at different pump fluences. (e) ASE peak intensity (circles) as a function of pump fluence for NPLc films. The solid lines are linear fits. (f) ASE thresholds as a function of NPLc film absorbance at pump wavelength (400 nm). The blue solid line is a fit according to the OG threshold model described in the main text.

S7. Optical gain mechanisms and threshold model

The absorption coefficient (α) at OG energy E_{OG} of the NPL ensemble, following Eq. 3 of the main text, is given by:

$$\alpha(E_{OG}) = N_{en}\sum_{i=0}^{N_s}[A_i(E_{OG}) + A_i^*(E_{OG})]N_i$$

$$\propto N_{en}A_T[\sum_{i=0}^{N_s}\left(\frac{N_s}{2} - i\right)N_i - 0.42N_sN_0 + 0.3N_1] \quad (S1)$$

Assumed a Poisson distribution of exciton numbers in NPLs, Eq. S2 becomes:

$$\alpha(E_0) \propto N_{en}A_T[\frac{N_s}{2} - \left[\sum_{i=0}^{N_s} iP_i + N_s\left(1 - \sum_{i=0}^{N_s} P_i\right)\right] - 0.42N_sP_0 + 0.3P_1] \quad (S2)$$

$P_n(m)$, the Poisson distribution possibility of finding NPLs with n excitons when the average number of excitons per NPL is m. Gain threshold is reached when $\alpha(E_0) = 0$. The solution to Eq. S2 under this condition gives the average number of excitons per NPL at OG threshold, $m_{th}(N_s)$, which is a function of N_s. We numerically solved Eq. S2 and all m_{th} values are listed in Table S2. We plotted $m_{th}(N_s)$ as a function of N_s in Figure S6, where we clearly observed a linear increase of m_{th} with N_s. The linear fit reveals a $m_{th}(N_s)/N_s$ ratio of 0.49±0.01.

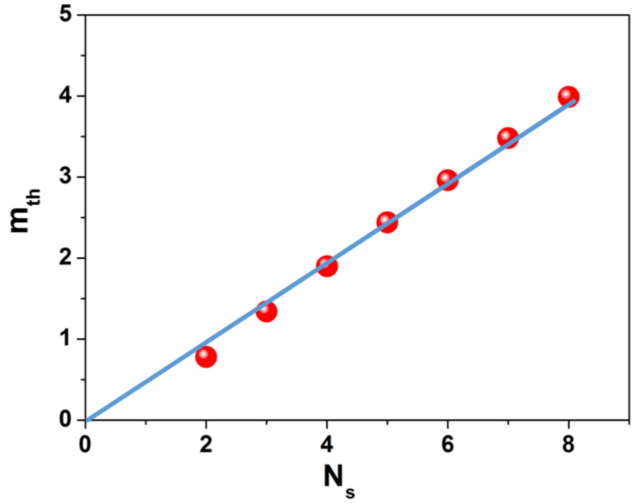

Figure S6. The average number of photons encountered with NPLs at OG threshold (m_{th}) as a function of N_s (red dots) and the linear fit (blue line).

Table S2. The average number of photons encountered with NPLs at OG threshold

N_s	m_{th}
2	0.78±0.01
3	1.34±0.01
4	1.90±0.01
5	2.44±0.01
6	2.96±0.01
7	3.48±0.01
8	3.99±0.01

The average number of photons in NPLs can be related to pump fluence I:

$$m = \frac{(1 - 10^{-\varepsilon z A_{QW} C_m L}) \frac{I}{h\nu}}{C_m N_A L} \quad \text{(S3)}$$

where $h\nu$ is pump photon energy, N_A is Avogadro constant, ε is molar absorption coefficient per unit NPL volume, z is NPL thickness, A_{QW} is NPL lateral area, L is light path of cuvette (1 mm), and C_m is molar concentration of NPL solution. From Eq. S3, we can obtain the pump fluence at OG threshold, i.e. the OG threshold I_{th}:

$$I_{th} = \frac{h\nu N_A}{\varepsilon z A_X}\frac{m_{th}(N_s)}{N_s}\frac{OD}{1 - 10^{-OD}} \quad (S4)$$

where $OD = \varepsilon z A_{QW} C_m L$ is optical density at pump wavelength. Substituting in $m_{th}(N_s)/N_s$ ~0.49, Eq. S4 can be rewritten as:

$$I_{th} = B\frac{OD}{1 - 10^{-OD}} \quad (S5)$$

We used Eq. S5 to fit the optical density dependent OG threshold and ASE threshold as shown in Figure 3c and 4d, respectively. The best fit gave the fitting parameter, B, as 88.9 μJ/cm^2 in Figure 3c and 89.7 μJ/cm^2 in Figure 4d.

Eq. S2 also predicts how optical gain increase with m. Shown in Figure S7a is a plot of normalized $-\alpha(E_0)$ as a function of m for different N_s (from 1 to 8). To compare with the pump fluence dependence of optical gain, we need to determine the average number of excitons per NPL, m, at any given pump fluence, using a well-established procedure. Following our previous TA studies on the same NPL samples,[4] only NPLs with single exciton remained at long delay time (t_L, 800-1000 ps). Therefore, the A exciton bleach at t_L (800-1000 ps) is proportional to the percentage of excited NPLs: 1-P_0(m). Following the same analyzing methods as reported in our previous work,[5] we define the normalized TA signal at t_L as:

$$\Delta S(\lambda, t_L) = \frac{\Delta Abs(\lambda, t_L)}{S(\lambda)} = 1 - P_0(m) = 1 - e^{-m} \quad (S8)$$

where λ is A exciton wavelength (~512 nm). These normalized TA signals represent the probability of finding excited NPLs in the solution sample. At high excitation intensities, when all NPLs were excited, $\Delta S(\lambda, t_L)$ approached one, from which the scaling factor $S(\lambda)$ was determined. According to Eq. S5, m is proportional to pump fluence: m=CI, and C is the photon encountering cross-section, which is proportional to lateral area (A_{QW}). Therefore, Eq. S8 became:

$$\Delta S(\lambda, t_L) = 1 - e^{-CI} \quad (S9)$$

As shown in Figure S7c to f, fitting $\Delta S(512nm, t_L)$ as a function of pump fluence I to Eq. S10 yields the value of parameter C of 0.023, 0.031, 0.040, and 0.045 cm^2/μJ for NPLa to d, respectively. Parameter C as a function of lateral area is plotted in Figure S7g, which confirms its linear increase with lateral area.

Our experimental OG amplitude as a function of m of all NPL samples were fitted

to Eq. S3 with N_s as the fitting parameter. The fitting results is plotted in Figure S7h. This plot agrees well with the measured OG saturation process and average exciton number at threshold (m_{th}) with different N_s.

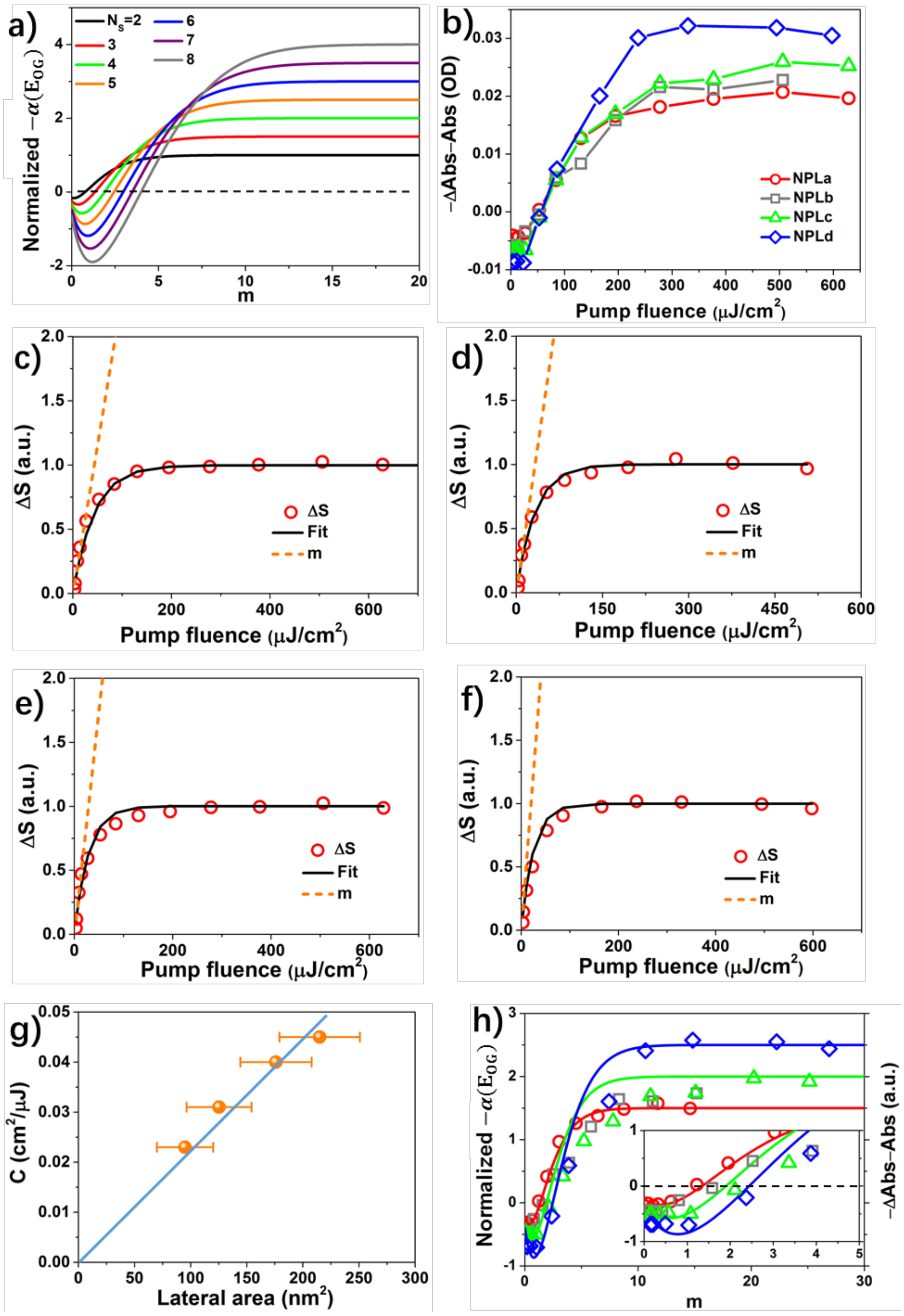

Figure S7. OG as a function of excitation fluence. (a) Simulated OG amplitude as a function of average number of excitons per NPL (m). (b) OG amplitude (at 3-4 ps) as a function of

pump fluence of NPLa to NPLd. (c-f) Normalized A exciton bleach amplitude at long delay time (800-1000 ps) as a function of pump fluence (open circles) and their fits (lines) for (c) NPLa, (d) NPLb, (e) NPLc, and (f) NPLd. (g) Photon encountering cross-section (C) of NPLs as a function of lateral area. Blue line is the linear fit. (h) Normalized OG amplitude of NPLa (red circles), NPLb (gray squares), NPLc (green triangles), and NPLd (blue rhombus) as a function of m. Solid lines are their fits according to Eq. S3: red line for N_S=3, green line for N_S=4, and blue line for N_S=5.

References

1. S. Ithurria, M. D. Tessier, B. Mahler, R. P. S. M. Lobo, B. Dubertret and A. Efros, *Nat. Mater.*, 2011, **10**, 936-941.
2. C. She, I. Fedin, D. S. Dolzhnikov, P. D. Dahlberg, G. S. Engel, R. D. Schaller and D. V. Talapin, *ACS Nano*, 2015, **9**, 9475-9485.
3. M. Olutas, B. Guzelturk, Y. Kelestemur, A. Yeltik, S. Delikanli and H. V. Demir, *ACS Nano*, 2015, **9**, 5041-5050.
4. Q. Li and T. Lian, *Nano Lett.*, 2017, **17**, 3152-3158.
5. Q. Li, Z. Xu, J. R. McBride and T. Lian, *ACS Nano*, 2017, **11**, 2545-2553.
